# Different Course of SARS-CoV-2 Infection in Two Adolescents With Other Immunosuppressive Factors

**DOI:** 10.7759/cureus.22710

**Published:** 2022-02-28

**Authors:** Karolina Kuczborska, Piotr Buda, Janusz B Książyk

**Affiliations:** 1 Pediatrics, Nutrition and Metabolic Disorders, Children’s Memorial Health Institute, Warsaw, POL

**Keywords:** immunodeficiency, sars-cov-2, hiv, down syndrome, covid-19

## Abstract

Even after two years of the Coronavirus Disease 2019 (COVID-19) pandemic, despite known risk factors, we are still unable to predict the severity of the infection in specific patients. Due to the contradictory data, the protective role of immunosuppression in preventing the severe course of the infection remains uncertain. Therefore, we want to discuss the influence of several immunosuppressive factors on the COVID-19 pattern in children, based on two case reports regarding 17-year-old boys with other immunosuppressive factors and a completely different course of the disease. The first patient suffered from AIDS, syphilis and primary central nervous system B-cell lymphoma, treated with radiotherapy. He experienced a light path of the infection, presenting only periodically appearing cough with no X-ray inflammatory changes. Nevertheless, due to the risk of severe COVID-19 and transient hypoxia, remdesivir was administered. He remained in a generally good condition and his follow-up did not reveal any noticeable complications. The second patient was characterised by Down syndrome, obesity, polyarteritis nodosa and chronic immunosuppressive therapy. He developed massive pneumonia, required treatment in the intensive care unit with the use of mechanical ventilation, remdesivir and anakinra. Despite the initial improvement of his general condition, including the degree of lung involvement and respiratory function, he developed an intracerebral haemorrhage, leading to brain herniation and ultimately death. In conclusion, HIV infection, oncological and immunosuppressive treatment do not seem to predispose to the severe course of COVID-19, whereas Down syndrome and obesity do.

## Introduction

Coronavirus Disease 2019 (COVID-19), a disease caused by Severe Acute Respiratory Syndrome Coronavirus 2 (SARS-CoV-2) infection, has been the most crucial health problem around the world for the last two years. Our knowledge about the infection, its spread, susceptibility, severity and treatment has changed a lot over this time. Compared to the first wave of the pandemic, we currently have vaccines that protect us against the severe course of the infection, as well as the first-line drugs effective in COVID-19 pneumonia. Nevertheless, despite the known risk factors, scientists and physicians are still unable to predict the severity of the infection in specific patients, who are often amazed by the seriousness or lightness of their course of the disease.

The vast majority of publications prove that immunocompromised patients, especially children, are characterised by a mild course of SARS-CoV-2 infection and therefore rarely require hospitalisation [1,2]. However, some researchers deny this statement and present a severe course of COVID-19 in this group without indicating any additional risk factors [3]. With this contradictory data, the protective role of immunosuppressants in avoiding the infection's severe course and its complications remains uncertain. Staying with this controversy, we would like to present two case reports of 17-year-old boys not vaccinated against COVID-19, with other immunosuppressive factors and with a completely different course of the disease.

## Case presentation

First case

A 17-year-old boy, previously diagnosed with AIDS with CD4 count 396/mL, primary central nervous system (CNS) B-cell lymphoma, secondary syphilis and suspected CNS syphilis, was admitted to our department during quarantine for radiotherapy. The patient was diagnosed with B-cell lymphoma and AIDS seven months before admission. During this period, the patient underwent chemotherapy, and the patient experienced bleeding in the CNS complicated by cardiac arrest and Rickham reservoir implantation. Three weeks before admission, he began radiotherapy with good tolerance. On the day of admission, a nasopharyngeal swab was collected from the patient and his mother to perform the reverse transcription polymerase chain reaction (RT-PCR) test for SARS-CoV-2. The test was positive for the mother and negative for the child. They were transformed to the COVID department, where the treatment was continued. During this stay, two sessions of radiation therapy to the CNS were performed without complications.

A day after oncological treatment, due to periodically appearing cough, a nasopharyngeal swab was collected from the patient to perform the RT-PCR test for SARS-CoV-2, which turned out to be positive. His chest X-ray revealed no inflammatory changes, and he had no dyspnoea or fever. Yet, due to the risk of severe COVID-19 and transient hypoxia, remdesivir was administered, and the treatment was continued for seven days. The patient remained in a good condition, coughed periodically and his blood oxygen saturation remained normal. Due to leukopenia, granulocyte colony-stimulating factor (G-CSF) was administered twice. After the end of the treatment, he was discharged home in a good condition, and his follow-up did not reveal any noticeable complications.

Second case

**Figure 1:**
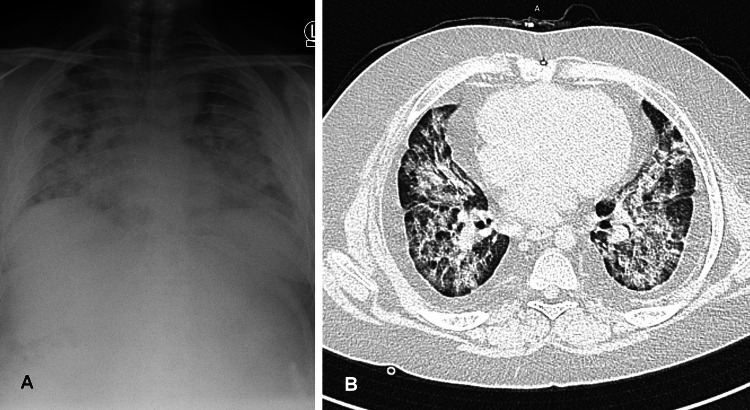
Radiological findings on chest X-ray and CT scan of the second patient (A) Chest X-ray, PA view: massive bilateral inflammatory changes. (B) Chest CT, transverse plane: lungs with reduced volume, extensive bilateral parenchymal and interstitial densities, multifocal ground-glass opacity. Changes are more intense in the right lung, involving approximately 60% of the lung parenchyma. CT: computerized tomography; PA: posteroanterior.

A 17-year-old boy with Down syndrome, obesity, childhood-onset polyarteritis nodosa (PAN), on chronic immunosuppressive therapy (methotrexate and methylprednisolone), after the correction of atrioventricular canal (AVC), was admitted to our department due to fever and decline in blood oxygen saturation. He was hospitalised in our department four weeks earlier due to massive pneumonia. COVID-19 was ruled out. The present admission was preceded by fever, cough and dyspnoea for three days. On admission, he presented with tachypnoea and respiratory effort and therefore required oxygen therapy at a flow of 7 L/min, at which the blood oxygen saturation level remained at 86-88%. His chest X-ray revealed massive bilateral inflammatory changes (Figure 1A), whereas computed tomography scan revealed multifocal ground-glass opacity and decreased lung volume, indicating COVID-19 (Figure 1B). He had significantly elevated inflammatory markers (C-reactive protein 14.4 mg/dL; procalcitonin 0.15 ng/mL; ferritin 5695.35 ng/mL), and his nasopharyngeal swab for SARS-CoV-2 was positive. Neither he nor his parents were vaccinated against COVID-19, despite being advised to do so.

He received meropenem, dexamethasone, remdesivir and low-molecular-weight heparin. Due to the symptoms of macrophage activation syndrome (MAS), hyperferritinaemia, leukopenia and the underlying chronic inflammatory disease (PAN), anakinra was administered. Because of the lack of improvement in the patient's general condition and respiratory parameters on high-flow nasal oxygen therapy, he was transferred to the ICU. There he required mechanical ventilation, sedation, flaccidity, anti-inflammatory (glucocorticosteroids and anakinra in reduced doses) and antihypertensive medications, as well as insulin infusion due to hyperglycaemia. The treatment administered in the paediatric ward was continued, including remdesivir, for seven days. Initially, the patient's general condition improved, including the degree of lung involvement and respiratory function. He was weaned from mechanical ventilation after one month. However, after this, the boy developed an intracerebral haemorrhage, leading to brain herniation and death.

## Discussion

From the beginning of the COVID-19 pandemic, a mild course of the disease was noticeable in children. The younger the child, the lower the risk of severe course, complications and hospitalisation. However, there was a concern for children with chronic illnesses, especially those receiving immunosuppressive drugs or undergoing chemotherapy. Further observations showed that these children are predominantly as mildly affected by COVID-19 as their healthy peers. As an excessive, tissue-damaging immune response causes a severe COVID-19 pattern, it is currently assumed that the state of reduced immunity in children is usually a protective factor. Nevertheless, it is still unclear whether this protective role differs in children with primary and secondary immunodeficiency and specific diagnoses or treatments.

In our case, the first patient had two immunosuppressive factors: AIDS and lymphoma accompanied by radio- and chemotherapy. The research results regarding the course of COVID-19 in children infected with HIV are scarce. Therefore, we need to focus on reports on adult patients. Several scientists have tried to answer the question of whether HIV status portends poor COVID-19 outcomes. Lesko and Bengtson claimed that people living with HIV (PLWH) are as vulnerable to SARS-CoV-2 infection as the standard population [4]. Simultaneously, Durstenfeld et al., in the study involving 220 PLWH from American hospitals, proved that HIV does not worsen the COVID-19 course, including mortality and adverse cardiac events [5]. However, Kanwugu and Adadi emphasised that the vast majority of described patients are on antiretroviral therapy (ART) and their viral replications are well controlled [6]. Therefore, these findings may not be similar among patients with high viremia, low CD4 cell count or other comorbidities. Our patient was not receiving ART before contracting COVID-19. Moreover, he had CNS B-cell lymphoma, drug nephropathy, cachexia and radiotherapy. Nevertheless, all these comorbidities did not alter his mild COVID-19 pattern. As for the beneficial effects of remdesivir in this group, there are limited data on its use in PLWH. However, taking into account the balance of risks and benefits, we decided on the drug administration. Nevertheless, its effectiveness cannot be confirmed based on a single case report.

The effect of the patient's lymphoma and the treatment against it on the course of SARS-CoV-2 infection seems to be better understood and less ambiguous than that of AIDS. Many studies on oncological paediatric patients confirmed that tumours do not impair benign COVID-19 patterns in this group. Moreover, radio- and chemotherapy can be continued during this period as they are well tolerated. Millen et al. from the United Kingdom and Ferrari et al. from Lombardia did not feel the need for any significant delays in oncological treatment if coexisting acute states did not require it [2,7]. Based on these reports, we also continued routinely both chemo- and radiotherapy in our centre, without observing increased complications regarding the oncological treatment as well as the course of acute infection.

Also, the impact of Down syndrome (DS) on the course of COVID-19 seems to be well understood, and reports about it are consistent. Several scientists have proved that children with DS are at higher risk of SARS-CoV-2 infection-related poor outcomes. They have a higher prevalence of respiratory syndromes, more often require hospitalisation and mechanical ventilation, are more vulnerable to complications and the course of their disease is associated with greater mortality [8,9]. There are several explanations for this phenomenon. First, immune dysregulation and specific anatomical airway features increase vulnerability to viral, respiratory infections [10]. Second, they are often characterised by comorbidities with a proven negative impact on the COVID-19 course, such as obesity, heart defects or hypothyroidism [8]. Finally, the increased number of 21st chromosomes entails substantial consequences. It contains the locus of the transmembrane serine protease 2 (TMPRSS2) gene, which is necessary for the viral entry into cells via an angiotensin-converting enzyme 2 (ACE2) receptor [8,11]. Moreover, four interferon (IFN) receptors are located on the 21st chromosome (IFNAR1, IFNAR2, IFNGR2 and IL10RB), and their overexpression leads to hypersensitivity of the IFN pathway in the pulmonary tissue and consequently increases the cytokine storm syndrome responsible for the pathogenesis of the severe COVID-19 pattern [12]. Summarising the impact of molecular factors, children with DS are more vulnerable to viral penetration and lung tissue inflammation caused by it. Due to all the above reasons, great emphasis is put on the validity of vaccinating children with DS against COVID-19.

Our second patient was also characterised by more than one immunodeficiency factor. He suffered from PAN treated with immunosuppressants. Unfortunately, only limited data are available about the course of COVID-19 in paediatric rheumatology patients. Present reports claimed that these patients do not have a propensity to a more frequent or severe SARS-CoV-2 infection [13,14]. Moreover, researchers emphasise that anti-rheumatic medication should be continued during this period since uncontrolled underlying disease and its high activity are crucial predictors of infection in these children [13,15]. Interestingly, some anti-rheumatic drugs and monoclonal antibodies are beneficial in the fight against COVID-19. The most promising therapies are anti-IL-1 and anti-IL-6 medicaments (anakinra and tocilizumab, respectively), which reduce the need for mechanical ventilation and decrease mortality in severe COVID-19 [16,17]. However, there are no clear-cut guidelines about using the abovementioned drugs under such conditions. Initially, our patient continued his basic immunosuppression scheme but after the deterioration of his general condition and development of MAS, a decision was made to administer him with anakinra, with which our hospital has more experience than with tocilizumab. Unfortunately, although it reduced the severity of inflammation, it did not prevent further complications, leading to death.

Apart from DS, obesity appeared to be the major contributor to the severity of COVID-19 in our second patient. Obesity is one of the best-proven risk factors for deteriorating the course of the disease and significantly increasing the risk of hospitalisation in the ICU in adults and children [18]. There are various causes and mechanisms of this phenomenon. First, obese patients are characterised by chronic subclinical inflammation, which is probably induced by adipokines produced in adipose tissue [19]. As severe COVID-19 is caused by the cytokine storm syndrome resulting in tissue damage, especially in the respiratory area, this pro-inflammatory state is an important trigger for pneumonia [18,19]. Second, it is proved that respiratory physiology is impaired in obese children [18]. Their haematosis is defective due to the adiponectin that damages pulmonary vascular endothelium [20]. This state, combined with the restriction of chest mobility caused by the pressure exerted by abdominal obesity, is deteriorating proportionally to the area of inflammation of the lung tissue. This reduces the blood oxygen saturation level and worsens the patient's general condition [19]. Finally, cardiovascular changes also play an important role. Obesity in any age group is often associated with higher blood pressure that may cause endothelial dysfunction, one of the major factors underlying the pathophysiology of COVID-19 [18].

## Conclusions

Although the majority of immunocompromised children are spared from the consequences of COVID-19, few individuals develop a more severe pattern of this disease. Unfortunately, it is still a matter of debate as to which diagnoses or treatments worsen the prognosis, and patients continue to surprise with a much heavier or lighter course of the disease than expected.

The impact of HIV infection is still controversial. In the case of remaining on antiretroviral therapy, with well-controlled viral replication and lack of comorbidities, HIV is not associated with adverse outcomes of COVID-19. Otherwise, the effects of the infection may be less favourable. However, this hypothesis is based on scarce data and requires further research, especially among children. With his mild COVID-19 course, the patient described by us is certainly a phenomenon and may not be an excellent example of a previously unknown population. On the other hand, it can be concluded that oncological and immunosuppressive treatment does not predispose to the severe course of COVID-19, whereas DS and obesity do. Therefore, patients with DS, especially those obese and with comorbidities, and their relatives should be strongly encouraged to get vaccinated. Moreover, the use of remdesivir among immunocompromised children may have a beneficial effect both on the treatment and prevention of the severe course of COVID-19 pneumonia. However, the latter conclusion requires confirmation in further studies.
